# Safety of Obstetric Ultrasound: Mechanical and Thermal Indexes—A Systematic Review

**DOI:** 10.3390/jcm13216588

**Published:** 2024-11-01

**Authors:** Antonia Varthaliti, Zacharias Fasoulakis, Vasilios Lygizos, Vasiliki Zolota, Maria Ioanna Chatziioannou, Maria Anastasia Daskalaki, George Daskalakis, Panos Antsaklis

**Affiliations:** 1 1st Department of Obstetrics and Gynecology, Alexandra General Hospital, National and Kapodistrian University of Athens, 11528 Athens, Greece; hzaxos@gmail.com (Z.F.); vlygizos@gmail.com (V.L.); vasoullou92@gmail.com (V.Z.); chatziioannou.marianna89@gmail.com (M.I.C.); anastasia.daskalaki00@gmail.com (M.A.D.); gdaskalakis@yahoo.com (G.D.); panosant@gmail.com (P.A.)

**Keywords:** ultrasound, mechanical, thermal, index, acoustic, output, safety, obstetrics

## Abstract

**Background/Objectives**: Obstetric ultrasound is one of the most commonly used imaging modalities during pregnancy to detect any fetal abnormalities. The aim of this systematic review was to appraise all available scientific literature and summarize current evidence regarding the safety of fetal ultrasound by using the thermal index (TI) and mechanical index (MI). **Methods**: We applied the PRISMA guidelines in order to prepare the review, and a 2-step process was performed in order to evaluate the available literature and decide which studies to be included. A thorough search of the Medline, Scopus, and Google Scholar databases was performed. Randomized and non-randomized studies were considered for review. The MI and TI were available in ultrasound machines after 1993; thus, studies before that year would not provide data on these two indexes. **Results:** A total of 21 studies were included in this review, including prospective, retrospective, cross-sectional, and survey-type studies. A common theme of the majority of the studies is the increased acoustic output available to the machines with time and the limited awareness of where the MI/TI indexes are from the operators. **Conclusions:** This review indicates that, while obstetric ultrasound is predominantly safe, there is a need for operators to consistently observe MI/TI indexes and adhere to the ALARA principle to minimize potential risks.

## 1. Introduction

Obstetric ultrasonography, or ultrasound (US), was developed and first used in the mid-1950s, and since then a tremendous growth in use and clinical capabilities has been observed [1]. Ultrasound devices are now commonly available and can provide diagnostic evaluations in the emergency department, outpatient setting, private office, or even private residential spaces and offer objective and precise measurements.

Fetal US exams should be performed when clinically indicated, by trained personnel, and by following the As Low As Reasonably Achievable (ALARA) principle regarding the acoustic output. Ultrasonography involves the use of sound waves, and there have been no reports of adverse fetal events as stated by the American College of Obstetricians and Gynecologists [2]. However, as a form of energy, ultrasound has the potential to have certain bioeffects on living tissues, with heating and cavitation being the two most common bioeffects [3,4].

In 1992, the U.S. Food and Drug Administration (FDA) raised the output levels of ultrasound machines to 720 mW/cm^2^ (from 94 mW/cm^2^), which theoretically can increase the fetal temperature by 2 °C but requires that the person performing the ultrasound is aware of any possible bioeffects the scan may be causing [2,5]. This was made possible by the output display standard (ODS), which exhibits in real-time the thermal index (TI) and mechanical index (MI) and gives the operator the opportunity to adjust the acoustic output when necessary in order to follow the ALARA principle [6].

The objective of this systematic review is to appraise all available scientific literature and summarize current evidence regarding the safety of fetal US by using the TI and MI.

This systematic review is registered with PROSPERO (CRD42023463770).

## 2. Materials and Methods

### 2.1. Definitions

Thermal energy exposure is assessed using the thermal index (TI), defined as the ratio of the emitted acoustic power to the power required to raise the temperature of tissue by 1 °C. The non-thermal biological effects of ultrasound imaging (e.g., pressure, cavitation) are assessed using the mechanical index (MI), defined as the maximum value of the peak negative pressure divided by the square root of the acoustic center frequency [7].

### 2.2. Study Design

This was a systematic review, and we applied the Preferred Reporting Items for Systematic Reviews and Meta-Analyses (PRISMA) guidelines [8]. An Institutional or Ethical Review Board was not required, as this systematic review collects publicly available data [9].

### 2.3. Literature Search and Data Collection

**Figure 1:**
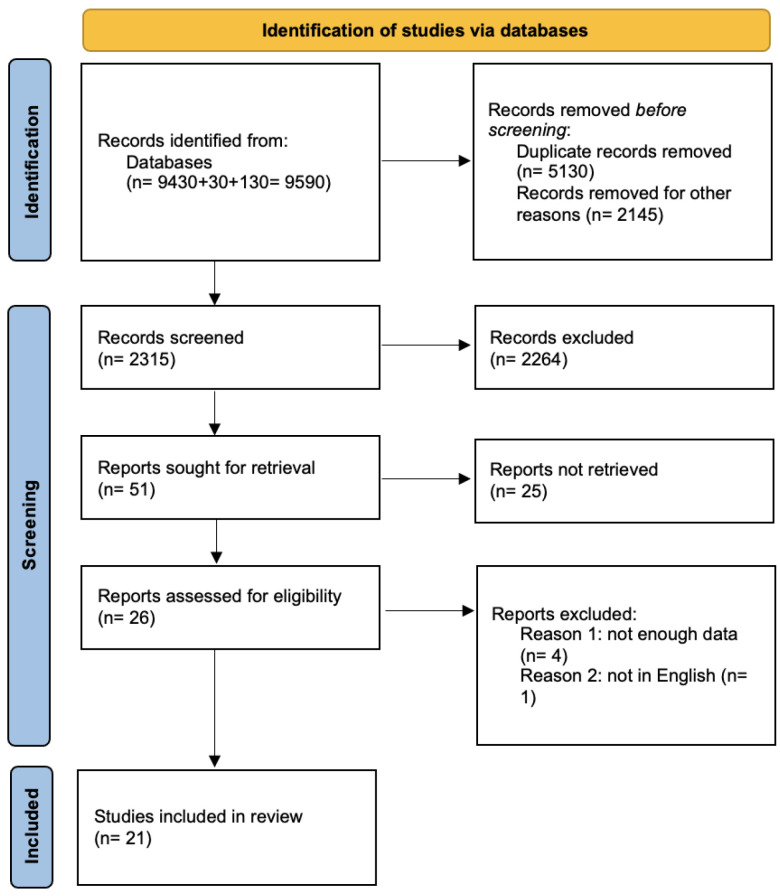
Search flow diagram based on PRISMA guidelines.

A thorough search of Medline, Scopus, and Google Scholar databases has been performed until 18 August 2023. The main search algorithm was common for the aforementioned databases and included the following terms: (safety) AND (obstetric) AND (ultrasound OR ultrasonography) AND (mechanical) AND (thermal) AND (index) AND (acoustic) AND (output). The PRISMA flow diagram displays the article assortment process (Figure 1).

The study selection was completed in two successive phases. First, all available studies that were initially identified were screened in order to see whether they satisfied the eligibility criteria. Second, duplicate publications were removed, and then the full text of the remaining articles was read in order to assess their eligibility.

### 2.4. Eligibility Criteria

Eligibility criteria were predetermined by the authors. Two independent reviewers agreed on the selection of the eligible studies and achieved consensus on which studies to include. Randomized and non-randomized studies involving human patients (not in vitro or animal studies), including prospective and retrospective studies, were considered eligible for this systematic review. In addition, studies that were written only in English were evaluated for this review.

Special consideration needs to be given regarding the date of the publications. As already mentioned, MI and TI indexes were available in ultrasound machines after 1993; thus, studies before that year would not provide data on these two indexes.

### 2.5. Assessment of Quality and Risk of Bias

For non-randomized studies, the Newcastle-Ottawa Scale tool was used to assess the quality of the data reported in these studies [10]. For randomized trials, Version 2 of the Cochrane risk-of-bias tool for randomized trials (RoB 2) was used to detect the risk of bias [11].

### 2.6. Statistical Analysis

No specific statistical analysis was performed for this particular systematic review since it did not involve a meta-analysis; thus, the aim was to describe the findings of each study and extract vital information that may be helpful for those performing obstetric US in their daily practice.

## 3. Results

### 3.1. Excluded Studies

A study investigating the use of a reference tool was not included as it had very few respondents, and hence, it would not offer considerable results [12]. An analysis from the Helsinki Ultrasound Trial regarding the possible effect of prenatal ultrasound in higher rates of non-right-handedness was not included as it did not provide detailed US data [13]. The study by Moderiano et al. was not included as it did not provide enough evidence regarding obstetric US knowledge [14]. We did not include the paper by Retz et al., as it was not clear if the measurements were made during obstetrical scanning [15]. Non-English studies were also excluded from further evaluation [16].

### 3.2. Included Studies

Twenty-one studies were included in total for this systematic review, and for each study, vital information regarding the design and main results is presented in Table 1 and is briefly described herein in reverse chronological order.

Fatahi Asl et al., in an observational study of 79 patients undergoing pregnancy scans, showed that Doppler studies were used mostly for these patients and did not show any significant difference among MI, TI, and scanning times compared to conventional scanning [17]. Of note, the mean MI was higher than the permitted level for the first trimester of pregnancy (1.22  ±  0.08). In addition, Drukker et al., in their prospective study of 637 patients in all three pregnancy trimesters, showed the highest Tib to be recorded for pulsed-wave Doppler mode in all trimesters [18]. Furthermore, in about 4% of the scans, the operators observed the bioeffect safety indices. Moreover, Mashiane et al. published a cross-sectional survey of 515 questionnaires that were distributed to participants of two national congresses in South Africa to evaluate the knowledge of operators regarding US safety [20]. Of these 515, analysis was performed in 121 questionnaires; a high rate of sonographers was aware of the ALARA principle, and there was a trend of increased awareness of ALARA with a higher number of scans. Flint et al. proposed the use of a lag-one coherence (LOC) metric that can identify the lowest MI value during real-time scanning while at the same time offering adequate image quality [21].

There have been limited investigations into the potential link between the acoustic power of obstetrical ultrasound and the subsequent development of autism spectrum disorders (ASD). The evidence on this topic is far from conclusive. One study of note is that by Rosman et al., which retrospectively examined the possible association between ultrasound use and autism development. In this study, 420 participants were identified, consisting of 107 patients with autism spectrum disorder (ASD), 104 controls with developmental delay, and 209 controls with typical development. The study found that fetuses with ASD were exposed to a greater depth of ultrasonographic penetration during the first and second trimesters compared with typically developing children and during the first trimester compared with developmentally delayed children. However, there were no significant changes observed in the mechanical index (MI) or thermal index (TI) values across the three categories. This study suggests that while there may be differences in ultrasound usage among children who develop ASD and those who do not, the mechanical and thermal indices—specifically, measures of ultrasound intensity and potential thermal effects—were not found to be significantly different among the groups [22].

Smarr et al. explored a possible association between MI/TI > 1 and neonatal anthropometry, extracting data from the NICHD Fetal Growth Studies–Singletons, a prospective cohort study that recruited 2334 non-obese pregnant women [7]. There was equal representation of non-Hispanic white, non-Hispanic black, Hispanic, and Asian pregnant women. In essence, there was no evidence of association of MI/TI > 1 with infants’ birth size or anthropometry; however, they do report upper/lower limb changes with sporadic occurrences of MI/TI > 1. Nemescu et al. compared two US scanners in first-trimester fetal heart scans in 552 fetal heart examinations [23]. Essentially, their study proves that newer generation US scanners can provide lower TI for gray-scale exam of the heart and ductus venosus interrogation and lower MI for gray-scale, color-mapped imaging of the heart and evaluation of the tricuspid flow. In another prospective observational study by Nemescu and colleagues for first-trimester heart scans in a sample of 303 fetuses, the authors provided insightful data regarding acoustic output values during the learning curve of a new US system [24]. They observed a significant decrease in MI from the color Doppler examination of the fetal heart, and both MI and TI of the pulsed-wave Doppler initially increased but then decreased at later stages of the learning curve. Martin et al. conducted a survey to evaluate the use of transvaginal US in the United Kingdom [25]. The majority of the respondents were sonographers who had high levels of understanding of the meaning of MI/TI, but almost 40% of them stated that they rarely monitor MI/TI. In another prospective study including 399 fetal heart scans, Nemescu and colleagues reported low TI values for color flow and PW Doppler exams but higher compared to gray-scale exams and that the safety indices were stable and constant, especially for color Doppler (TI), tricuspid flow (MI) and ductus venosus assessment (TI, MI) [26]. Bromley et al. retrospectively evaluated 100 submissions from operators preparing for credentialing [27]. Around 20% of the operators had a TI > 1.0 (up to 1.6), indirectly showing a lack of awareness regarding the ALARA principle. Cibull et al. collected 124 submissions to the FDA regarding safety data during US fetal scans from 1984 until 2010 [28]. The evidence supports the increased acoustic output as years pass and new US machines develop, as well as a high TIb, especially during pulsed-wave Doppler scanning. Houston et al., in a survey of 165 obstetric–gynecology residents and maternal–fetal–medicine fellows, showed that training is extremely important, and their knowledge improved while they were advancing in their training [29]. Akhtar et al. provided a self-administered questionnaire at a national radiological congress in Pakistan, and even though around 33% of respondents knew what MI/TI was, less than 15% knew where to find these indexes at their respective US machines [30].

Finally, multiple studies by Sheiner and colleagues provide important evidence regarding the safety of the acoustic output indices during fetal scanning [3,5,31,32,33,34]. Of note, the comparison between 2D versus 3D/4D US scans did not show any significant changes in mean TI values during B-mode studies.

## 4. Discussion

The question of whether ultrasound, specifically its acoustic power, might be related to the development of ASD is not just a matter of direct cause-and-effect. Multiple factors, both genetic and environmental, contribute to the onset of autism. The broader scientific consensus, based on the current evidence, is that there is not a direct link between prenatal ultrasound and the development of autism. The American College of Obstetricians and Gynecologists has stated that there are no confirmed adverse fetal effects from diagnostic ultrasound used in standard examinations. The FDA also emphasizes the safety of ultrasound when used appropriately.

The study by Rosman et al. took a closer look at this potential relationship by analyzing parameters such as depth of ultrasonographic penetration, mechanical index (MI), and thermal index (TI) [22]. While they found that fetuses with ASD were exposed to a greater depth of ultrasonographic penetration during the first and second trimesters compared to typically developing children, and during the first trimester compared with developmentally delayed children, there were no significant differences observed in the MI or TI values among the groups. This suggests that while there may be differential ultrasound practices or variances based on other underlying factors, the acoustic power as indicated by MI and TI—which represent the mechanical and thermal aspects of ultrasound exposure, respectively—were not distinct among the groups.

It is essential to put the findings of individual studies into the larger context of scientific consensus. A previous randomized trial by Newnham et al. has shown that intensive ultrasound imaging during pregnancy might lead to intrauterine growth restriction, compared to only one ultrasound scan [35]. However, the primary point of this study was different: to measure whether intensive US imaging leads to better outcomes. In addition, the study dates back to 1993; since then, the safety and technical characteristics of the available US devices have considerably improved. The vast majority of research in the field has found no direct link between routine prenatal ultrasound and the development of autism. Major medical bodies, such as the American College of Obstetricians and Gynecologists, maintain that when used appropriately, diagnostic ultrasounds pose no confirmed adverse fetal effects. Similarly, the U.S. FDA emphasizes the safety of ultrasound when applied appropriately, suggesting that any potential risk would likely arise from inappropriate or excessive use rather than standard diagnostic procedures.

While the study by Rosman et al. sheds light on a potentially intriguing area of research, it is crucial to acknowledge its limitations [22]. Retrospective studies inherently have potential biases, and while they can identify associations, they cannot definitively establish causality. Furthermore, it is equally important to consider potential confounding factors that might influence the results. For instance, the reason for the increased depth of ultrasonographic penetration in fetuses with ASD needs further exploration. Was it due to specific maternal or fetal indications that could be related to ASD development?

The intricacies of autism’s etiology mean that isolating any single factor is challenging. To conclusively determine any link between obstetrical ultrasound and ASD, more comprehensive, prospective studies that control for potential confounders and biases would be necessary. Until such studies are undertaken and replicated, it remains imperative for both the scientific community and the general public to approach this potential link with measured caution and a reliance on the broader body of evidence.

Training current and future obstetric US operators is of utmost importance, and attention should be implemented while designing training curricula. Although it is worth considering that depending on the circumstances (i.e., emergencies, out-of-hours, etc.), ‘proper’ training might not be provided, it is still valuable time for the trainee, and every possibility should be offered in order for the trainee to gain ‘hands-on’ training. In addition, while learning how to scan and detect anomalies is considered prime for every trainee, time should be spent for the trainees to learn the physics of the US machines and understand the principles besides the measurements they are taking. Furthermore, we need to consider that training might be different in different parts of the world. Resources can differ quite significantly, and this may lead to discrepancies in patient care. Thus, every attempt should be made to provide appropriate and unanimous training available to both physicians and sonographers.

When measuring the MI/TI indexes, the operator needs to be vigilant not to exceed certain levels and be able to correlate the MI/TI levels with the pregnancy’s trimester. It is true that during the first trimester that organogenesis occurs, MI/TI levels should be kept low, but that does not necessarily mean that these levels should be higher in subsequent trimesters. In addition, after the first 10 weeks of pregnancy, bone TI is important since ossification occurs at that stage, and vigilance is also necessary.

*Strengths and limitations of our study.* We undertook an exhaustive literature review, and we have provided all available data regarding the use of MI and TI during fetal US scanning. However, certain biases that may arise during the primary studies may not have been recognized and, as such, weaken the results of the present systematic review. Furthermore, we must acknowledge the lack of large-scale or longitudinal randomized controlled trials, and we also need to take into consideration the heterogeneity of the included papers, which could have a negative impact on our results. Nonetheless, we provide a balanced review of the available literature and offer an outlook on present-day procedures.

*Future research directions*. The safety of obstetric US is of paramount importance, as more and more women will be undergoing US scanning in the future. While it is expected that healthcare providers need to keep an advanced level of US knowledge and practice safety at all times, it is also expected that companies manufacturing US machines should also, in cooperation with scientists, develop novel devices and software that lead to safer scanning machines.

## 5. Conclusions

The findings of the present systematic review suggest that obstetric ultrasound is safe and that the operators performing the procedure are aware of the potential hazards of unnecessary long scanning times. Understanding and recognizing mechanical and thermal indexes should be an essential part of ultrasound training, and every effort should be made in order to abide by the ALARA principle.

## Figures and Tables

**Table 1:** Main characteristics of the included studies.

Author (Year)	Type of Study	Number of Cases	Points to Consider
Fatahi Asl et al. (2021) [17]	Observational	79	- Compared pregnancy and non-pregnancy values - Pregnancy check-ups at weeks 5–8 - Mean scan duration 10.98 ± 6.88 min - Mean TI was 0.33 ± 0.27 - Mean MI was 1.14 ± 0.13
Drukker et al. (2020) [18]	Prospective	637	- Scans were performed in all three pregnancy trimesters - PW Doppler had the highest TIb - Good adherence to thresholds - Operators do not check the bioeffect indicators when scanning
Wasickanin et al. (2020) [19]	Cross-sectional survey	138	- Assess knowledge of US safety - 42% of initial respondents completed the post-intervention survey - Intervention performed after the initial survey showed increased awareness of the respondents regarding safety indices
Mashiane et al. (2019) [20]	Cross-sectional survey	515	- 515 questionnaires distributed to 2 national congresses - Aim of the study is to evaluate knowledge of operators regarding US safety in South Africa71% ObGyn; 19% sonographers; 7% GPs; 3% MFM - 77% familiar with TI; 61% familiar with MI; 50% familiar with ALARA
Flint et al. (2018) [21]	Prospective	7	- Volunteers in second-trimester singleton pregnancies - Aim of the study was to test the lag-one-coherence MI metric for acquiring ALARA in real-time scanning - 80% of the time, ALARA MI was lower than the metric
Rosman et al. (2018) [22]	Retrospective	420	- Aim of the study was to assess prenatal US as a possible contributor to ASD - 107 patients with ASD, 104 controls with developmental delay, and 209 controls - Mean depth of penetration was higher in the ASD group in all trimesters - No significant effect of TI/MI values
Smarr et al. (2018) [7]	Prospective	2334	- TI or MI > 1 showed no effect on infants’ birth size or anthropometry - Women with ≥2 US scans with TI > 1 had neonates with small mid-upper arm and mid-upper thigh and had larger abdominal flank, anterior thigh, and subscapular skinfolds - Women with ≥2 US scans with MI > 1 had neonates with small mid-upper thigh, upper thigh length, and anterior thigh skinfold - Non-Hispanic Black had TI > 1 (36%) - Non-Hispanic White had MI > 1 (42%) - Women with ≥1 elevated (TI > 1) US scans (vs. women who never had elevated TI) had neonates with significantly smaller mid-upper arm circumferences and mid-upper thigh circumferences - Women with TI > 1 (vs. women without elevated TI) had neonates with larger abdominal flank skinfolds, subscapular skinfolds, anterior thigh skinfolds, upper arm lengths, upper thigh lengths
Nemescu et al. (2015) [23]	Prospective	552	- Fetal cardiac scans only - First trimester scans only - Compared 2 US scanners (Voluson 730 vs. E8, GE Healthcare, Austria) - Newer US machine provides lower TI for gray-scale exam of the heart, ductus venosus, and lower MI for gray-scale, color mapper imaging, and tricuspid flow evaluation
Nemescu et al. (2015) [24]	Prospective	303	- First-trimester heart scans - Evaluation of MI and TI values during the learning curve - MI decrease from the color Doppler - TI and MI from the PW Doppler of the tricuspid flow initially increased and then stabilized - TI and ductus venosus assessment was stable throughout the learning cure - Number of scans needed to achieve a high level of scanning = 140
Martin et al. (2015) [25]	Survey	294	- Investigate the safe performance of transvaginal US in the UK - 94% were sonographers - High levels of understanding of the meaning of MI/TI - Less than 15% always monitored MI/TI - Less than 15% that never monitored MI/TI
Nemescu et al. (2015) [26]	Prospective	399	- Fetal US cardiac exams in the first trimester - Low TI values for color flow and PW Doppler exams, but higher compared to gray-scale exams - Lower TI values from PW Doppler of ductus venosus versus tricuspid flow exam
Bromley et al. (2014) [27]	Retrospective	100	- Aim of the study was to evaluate ALARA compliance by credentialed operator scans performed at weeks 11–14 - 20% of operators had TI > 1.0 (~1.6 in some cases) - Registered ObGyn sonographers had significantly lower TI levels consistently (≤0.5)
Cibull et al. (2013) [28]	Survey	124	- FDA-received submissions dating from 1984–2010 - TIb values have increased exponentially time-wise (mean value of 0.3 compared to 0.19 ± 0.19)
Houston et al. (2011) [29]	Survey	165	- Electronic survey for ObGyn residents (n = 67) and MFM fellows (n = 92) - 11% of residents and 23% of fellows use MI/TI - >70% of both residents and fellows can appreciate the potential thermal injury - Fellows’ knowledge improved as training evolved
Akhtar et al. (2011) [30]	Survey	306	- Questionnaires handed over at a national congress in Pakistan - 55% consultant radiologists; 30% radiology residents; 10% sonographers; 15% others - 33% familiar with MI/TI - 13% knew where to find MI/TI in the scanner
Sheiner et al. (2009) [3]	Prospective	50	- First trimester NT scans - Mean scan duration 11.6 ± 4.2 min - Mean TI was 0.2 ± 0.1 - Mean MI was 1.1 ± 0.1
Sheiner et al. (2007) [31]	Prospective	63	- Second and third-trimester scans only - Mean scan duration 17.6 ± 8.6 min - CD and PW Doppler can have high TI levels (0.8 ± 0.1; 1.5 ± 0.5, respectively) compared to B-mode (0.3 ± 0.1) - MI levels were higher with B-mode and CD compared to PW Doppler (1.1 ± 0.1; 1.0 ± 0.1; 0.9 ± 0.2, respectively)
Sheiner et al. (2007) [32]	Survey	75	- Questionnaires handed over in meetings - 82% ObGyn; 16% MFM - 32% familiar with TI; 22% familiar with MI; 21% knew where to see MI/TI on the screen - No difference between physicians and non-physicians regarding knowledge and familiarity with MI/TI
Sheiner et al. (2007) [33]	Prospective	52	- First trimester scans - Mean scan duration 8.1 ± 1.4 min - Mean TI was 0.2 ± 0.1 - Mean MI was 0.9 ± 0.3
Sheiner et al. (2007) [34]	Prospective	40	- Comparison of 2D versus 3D/4D US scans - Mean scan duration 20.1 ± 9.9 min - Mean TI during 3D/4D examinations comparable to the TI during B-mode - MI significantly higher in 3D compared to B-mode and 4D
Sheiner et al. (2005) [5]	Prospective	37	- 11 first-trimester; 14 s-trimester; 12 third-trimester scans - Mean scan duration (1st: 8.9 min; 2nd: 31.8; 3rd: 16.3 min) - MI significantly increased across trimesters - No significant change was seen with TI across trimesters

## Data Availability

No new data were created or analyzed in this study. Data sharing is not applicable to this article.
